# Mechanistic perspectives on antimalarial agents: from FDA-approved drugs to next-generation candidates

**DOI:** 10.1039/d5ra08585c

**Published:** 2026-04-17

**Authors:** Monika, Bhawana Sharma, Satish Kumar Awasthi

**Affiliations:** a Chemical Biology Laboratory, Department of Chemistry, University of Delhi Delhi-110007 India satishpna@gmail.com +91-9582087608; b Department of Applied Sciences, The NorthCap University Gurugram-1220017 India

## Abstract

Malaria is a major global health challenge, demanding continued innovation in drug discovery and development. This review gives a comprehensive overview of FDA-approved antimalarial drugs and emerging clinical candidates, focusing their chemical structures, mechanisms of action, and molecular targets such as PfATP4, DHFR, DHODH, and PfCRT. The discussion showcases structure–activity relationships, mechanisms underlying drug resistance, and recent advances in structure-guided design of next-generation antimalarials. The review also summarizes the year of approval, mechanistic class, and synthetic origin of key therapeutic agents. Moreover, novel molecules currently in preclinical and clinical trials are discussed in the context of their mode of action, efficacy, and potential for overcoming resistance. Collectively, this article bridges medicinal chemistry insights with biological mechanisms, outlining future directions in the rational design of potent, resistance-resilient antimalarial drugs.

## Abstract 2

Overview of antimalarial drug classes organized by mechanism, including heme, PfATP4, folate, and mitochondrial inhibitors, showcasing representative drugs and next-generation candidates targeting resistance pathways.

## Graphical abstract

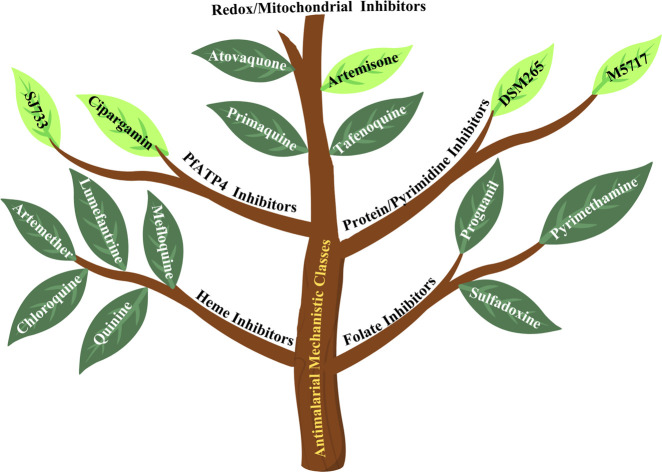

## 1 Introduction

The term “malaria” originates from the Italian phrase “mal'aria,” meaning “bad air,” reflecting its historical connection to swampy regions. Malaria is a vector-borne disease caused by *Plasmodium* parasites, which infect a wide range of hosts, including mammals, birds, and reptiles. Of the over 200 known *Plasmodium* species, five—*P. falciparum* (the most severe), *P. vivax*, *P. malariae*, *P. ovale*, and *P. knowlesi*—cause malaria in humans.^1^

Globally, malaria remains a significant public health concern, with an estimated 263 million cases and 597 000 deaths recorded in 2023. Africa bears the greatest burden, accounting for 94% of cases and 95% of deaths. However, substantial progress has been achieved since 2000, with over 2.2 billion cases and 12.7 million deaths averted. Despite these achievements, vulnerable populations, such as pregnant women in high-transmission regions, continue to face significant risks.^2^

**Fig. 1:**
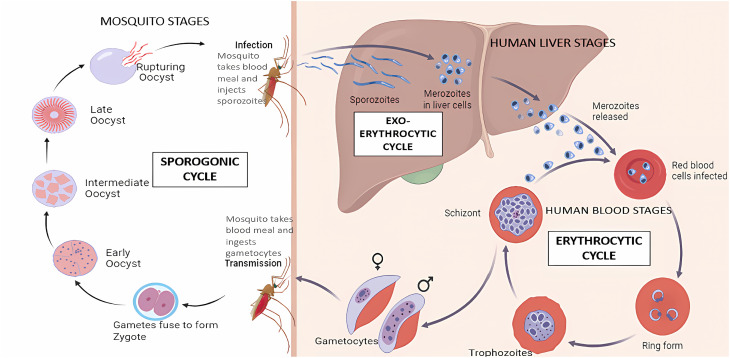
Life cycle of malaria. Created with https://www.biorender.com/and adapted from ref. 4.

*Plasmodium* transmission begins when an infected mosquito injects sporozoites into the human bloodstream. These sporozoites travel to the liver, where they infect hepatocytes, and multiply into merozoites. The infected liver cells rupture, releasing merozoites into the bloodstream, where they invade red blood cells (RBCs) and cause symptoms like fever and chills. Some merozoites develop into gametocytes, which are ingested by mosquitoes during a blood meal. Inside the mosquito, gametocytes mature, fertilize, and produce sporozoites that migrate to the mosquito's salivary glands, completing the transmission cycle (Fig. 1).^3^

**Fig. 2:**
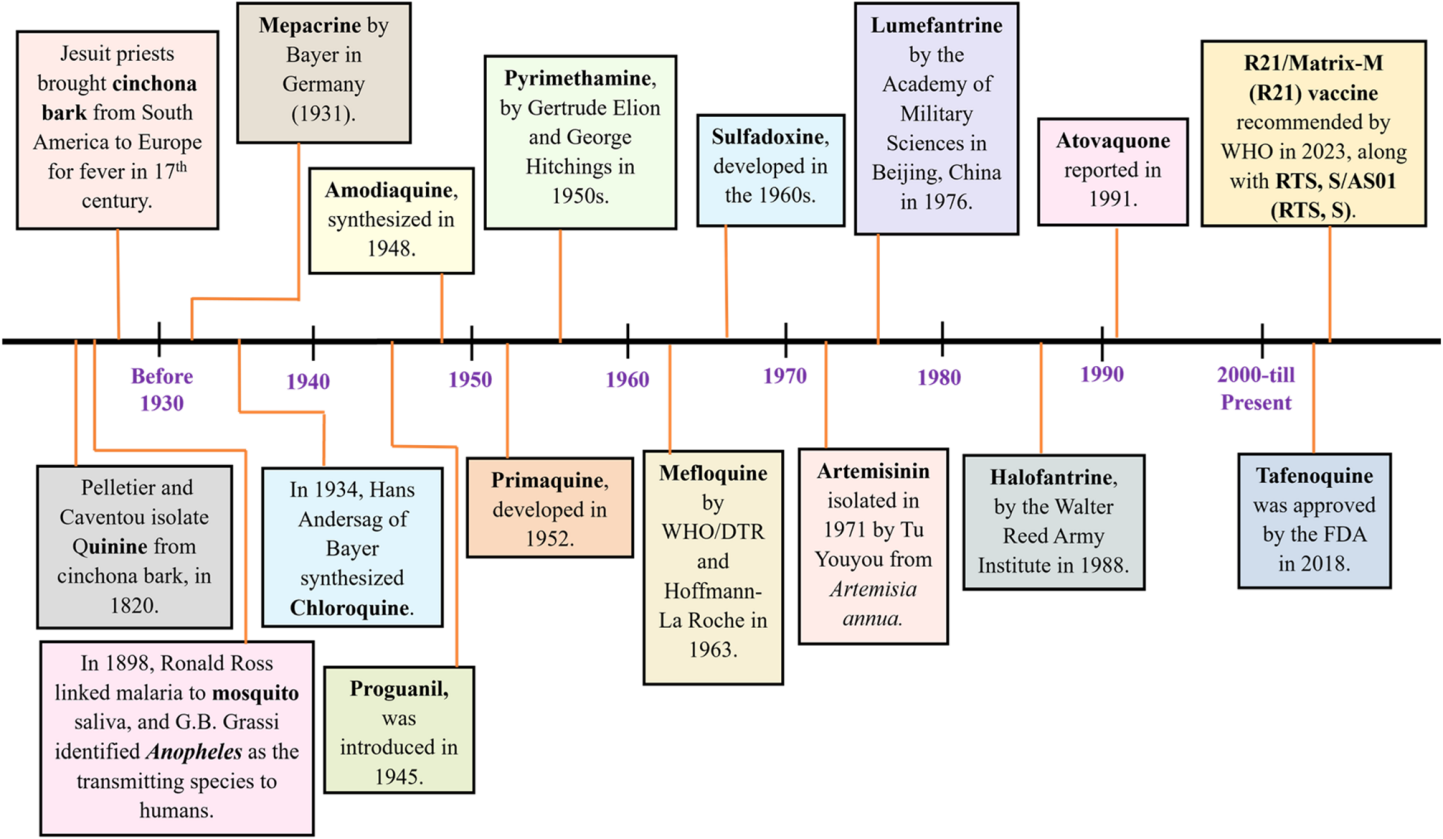
Landmark events in the discovery of antimalarial medicines.^2,5–10^

Recent progress in malaria treatment includes repurposed drugs like methylene blue with primaquine, fosmidomycin with piperaquine, and rosiglitazone as adjunctive therapy. New drug candidates, including M5717, MMV253, (+)-SJ733, UCT943, and MMV048, target specific parasite mechanisms. Promising clinical candidates such as P218, OZ439, KAE609, and DSM265 also showing potential. Despite these advancements, resistance, especially to artemisinin-based combination therapies (ACTs), remains a challenge, highlighting the need for novel therapies. The complexity of the *Plasmodium* life cycle poses additional obstacles to drug development, while issues like toxicity, delivery inefficiencies, and high costs hinder scalability. Current research focuses on single-dose cures, transmission-blocking drugs, anti-relapse therapies, and innovative combinations to overcome resistance and improve treatment outcomes.^5^Fig. 2 illustrates key events in antimalarial drug discovery.

This review provides an overview of currently FDA-approved antimalarial drugs, including their mechanisms of action, limitations, and therapeutic use, as shown in Table 1. Additionally, promising antimalarial compounds in advanced clinical trials and recent advancements are showcased in Table 2.

**Table 1:** FDA-approved antimalarial drugs

Generic name	Trade name	Year of FDA approval
Chloroquine	Aralen	1949 (ref. 10)
Primaquine	Primaquine phosphate	1952 (ref. 10)
Hydroxychloroquine	Plaquenil	1955 (ref. 11)
Sulfadoxine–Pyrimethamine	Fansidar	1983 (ref. 6)
Mefloquine	Lariam	1989 (ref. 10)
Atovaquone–Proguanil	Malarone	2000 (ref. 10)
Quinine	Qualaquine	2005 (ref. 6)
Artemether–Lumefantrine	Coartem	2009 (ref. 12)
Tafenoquine	Krintafel	2018 (ref. 10)
Intravenous Artesunate	Artesun	2020 (ref. 13)

**Table 2:** Promising synthetic antimalarial drug candidates that are currently under development, along with their structures, mechanism of action, safety profiles, and clinical status

Drug name	Structure	Class	Phase	Mechanism of action	Safety and tolerability
SJ733	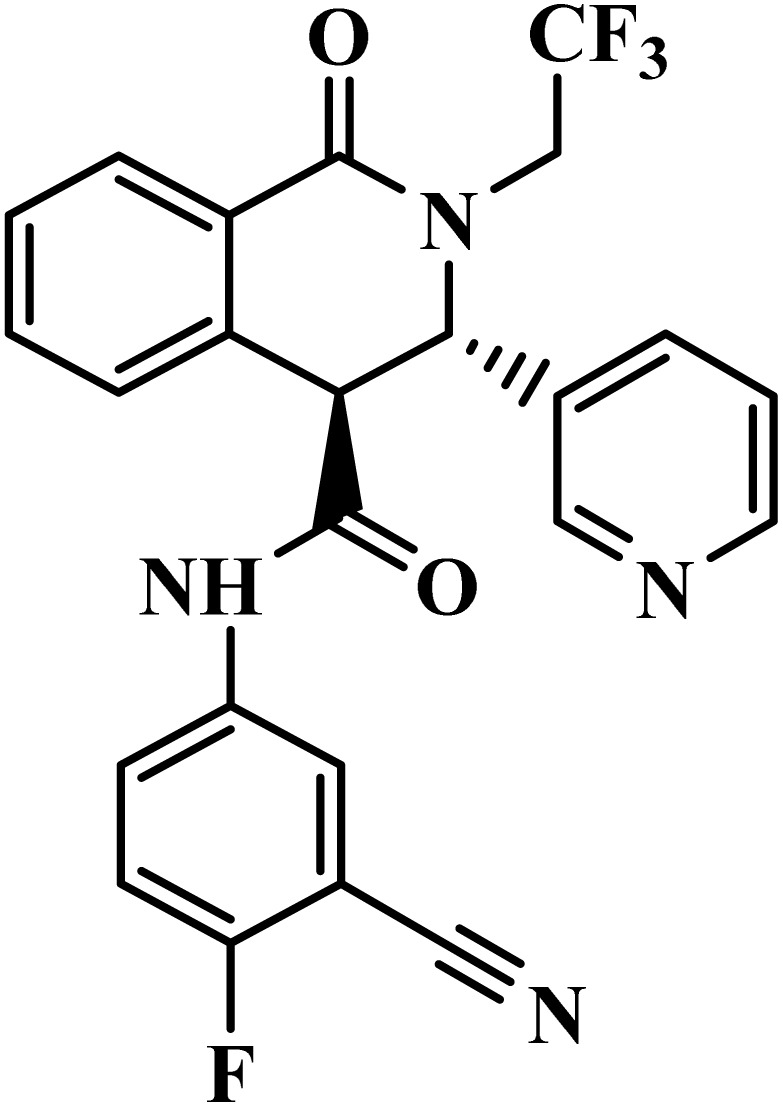	Dihydroisoquinolone	II	Disrupts the ATP4 function of *P. falciparum*^49^	(1) No significant toxicity observed in human trials (75–1200 mg)^50^
					(2) Rapid metabolism with no harmful accumulation^50^
					(3) Mild paraesthesia, occasional proteinuria, and low leucocyte count reported^50^
					(4) Advanced to Phase II clinical trials with no major adverse reactions^51^
AQ13	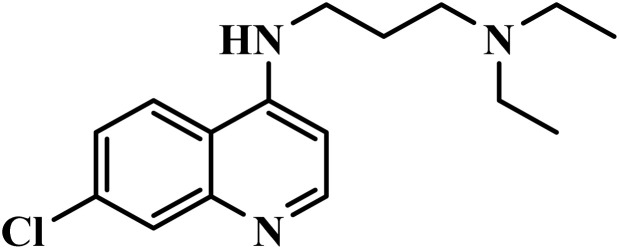	4-Aminoquinoline	II	The mechanism of action has not yet been evidenced^52^	(1) No organ toxicity at doses up to 1750 mg^53^
					(2) Mild adverse effects: Headache, nausea, diarrhea, pruritus^53,54^
					(3) Minimal QTc prolongation *vs.* chloroquine^53,54^
					(4) Comparable safety profile to chloroquine^51^
Cipargamin	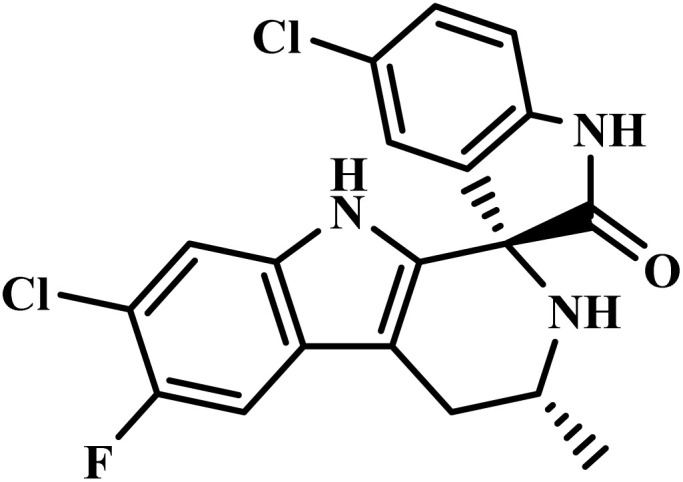	Spiroindolone	II	Blocks mosquitos' transmission and inhibits *Pf*ATP4 (ref. 55)	(1) Low cardiotoxicity risk (weak hERG binding)^56^
					(2) Mild GI/genitourinary adverse effects at higher doses^57^
					(3) Well tolerated up to 300 mg kg^−1^ in volunteers^57^
					(4) Safe in combination with piperaquine^51^
Imatinib	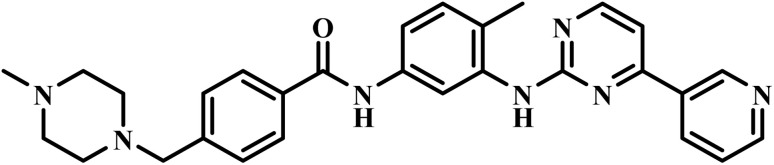	2-Phenylamino- pyrimidine	II	Inhibits the tyrosine phosphorylation of protein band 3(ref. 51)	(1) Risk of drug interactions *via* CYP3A4 and CYP2C8 metabolism^58^
					(2) Rifampicin decreases effectiveness; CYP3A4 inhibitors increase bioavailability^59,60^
					(3) Reported adverse effects: Asthenia, edema, diarrhea, muscle cramps, skin rash^61–63^
					(4) Rare but serious events include angina and heart failure^61–63^
M5717	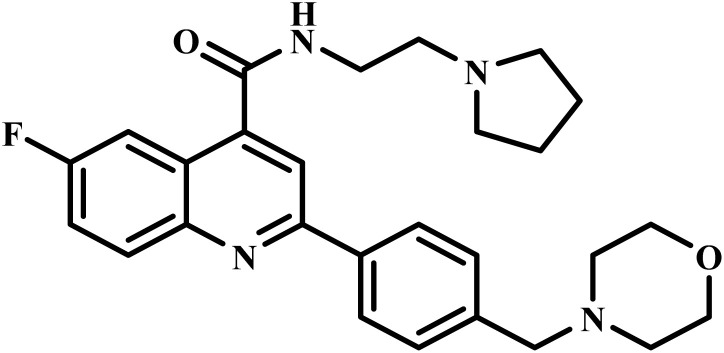	Quinoline diamine	II	Inhibits the protein synthesis of *P. falciparum* through disruption of the function of eukariotic translational elongation factor 2 (PfeEF2)^64^	(1) Non-toxic to human cells at therapeutic concentrations^65,66^
					(2) Minimal side effects reported in human studies.^64,66^
					(3) Favorable pharmacokinetics with high oral bioavailability and long half-life^5,67^
					(4) Good solubility and low protein binding^65^
Methylene blue	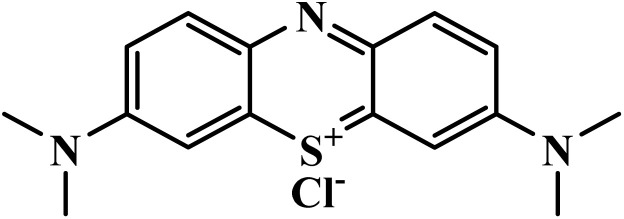	Phenothiazine	II	Inhibits the glutathione-dependent degradation of haem^68^	(1) Mild hemoglobin reduction in G6PD-deficient individuals^69^
					(2) Green–blue urine discoloration due to renal excretion^51^
					(3) Mild gastrointestinal and urogenital symptoms^51^
					(4) Generally safe in adults and children, including those with G6PD deficiency^70^
Sevuparin	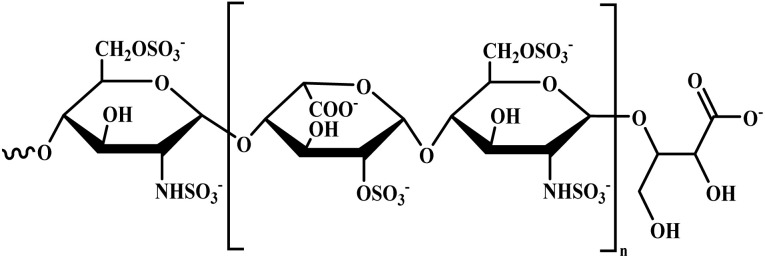	Acid polysaccharide	II	Prevents rosetting and cytoadherence of infected RBCs to the endothelium by binding to the DBL1α domain of PfEMP1 (ref. 51)	(1) No significant toxicity reported^51^
					(2) Safer than heparin due to absence of antithrombin activity^51^
Rosiglitazone	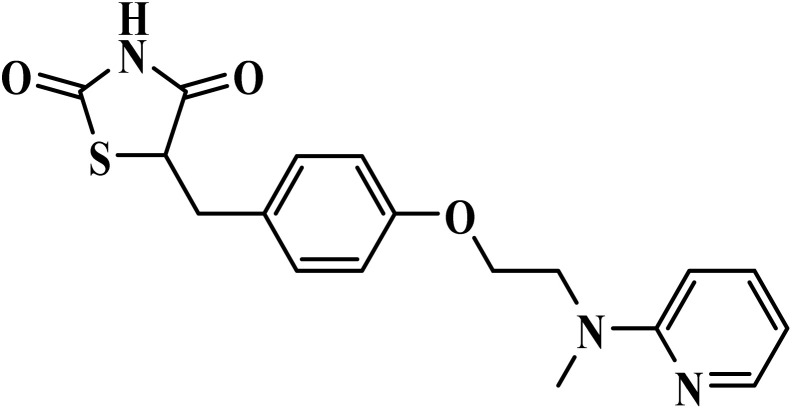	Thiazolidinedione	II	Facilitates macrophages phagocytosis of *P. falciparum* parasitized erythrocytes, and reduces induced parasites secretion of pro-inflammatory cytokines by monocytes and macrophages^71^	(1) No significant toxicity reported in malaria studies^72^
					(2) Does not interfere with immune response or artesunate pharmacokinetics^72^
					(3) Safe in adults and children (<12 years) in clinical trials^72^
DSM265	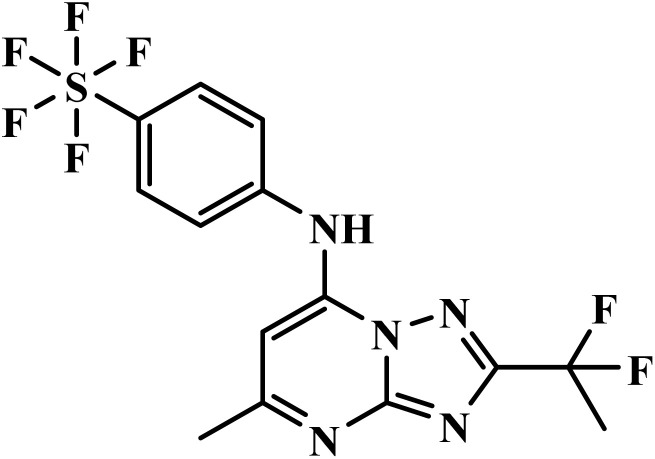	Triazolopyrimidine	II	Selectively blocks dihydroorotate dehydrogenase enzyme of *plasmodium* parasites^51^	(1) Non-genotoxic with low risk of CYP-mediated hepatotoxicity^73,74^
					(2) Generally well tolerated with a favorable safety profile in human trials^73^
					(3) Thrombocytopenia and elevated reticulocytes reported in some participants with hemoglobinopathy^73^
					(4) No hemolytic anemia observed in G6PD-deficient mouse models^73,74^
Ferroquine	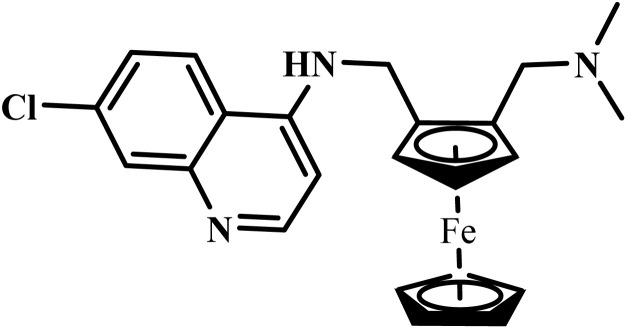	Ferrocene	II	Inhibits formation of hemozoin and generates reactive oxygen species^51^	(1) Potential hepatotoxicity (elevated liver enzymes) and QT interval prolongation reported^75^
					(2) Common adverse effects: Nausea, vomiting, dizziness, and anemia^75^
					(3) Generally well tolerated in adults and children^75^
Aretefenomel	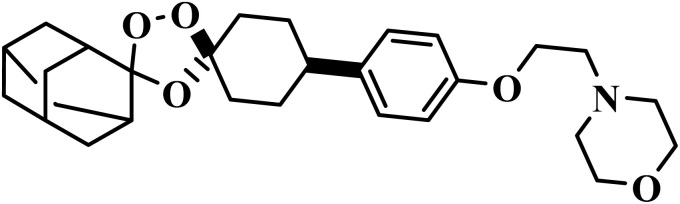	1,2,4-Trioxolane	III	Disrupts the haemoglobin digestion by the parasite^76^	(1) Low toxicity with lower embryotoxicity compared to artemisinins^77^
					(2) Common mild effects: diarrhea, nausea, headache, flushing, dyspepsia, vasovagal syncope^78^
					(3) Safe up to 800 mg day; potentially safer in pregnancy^78^
Artemisone	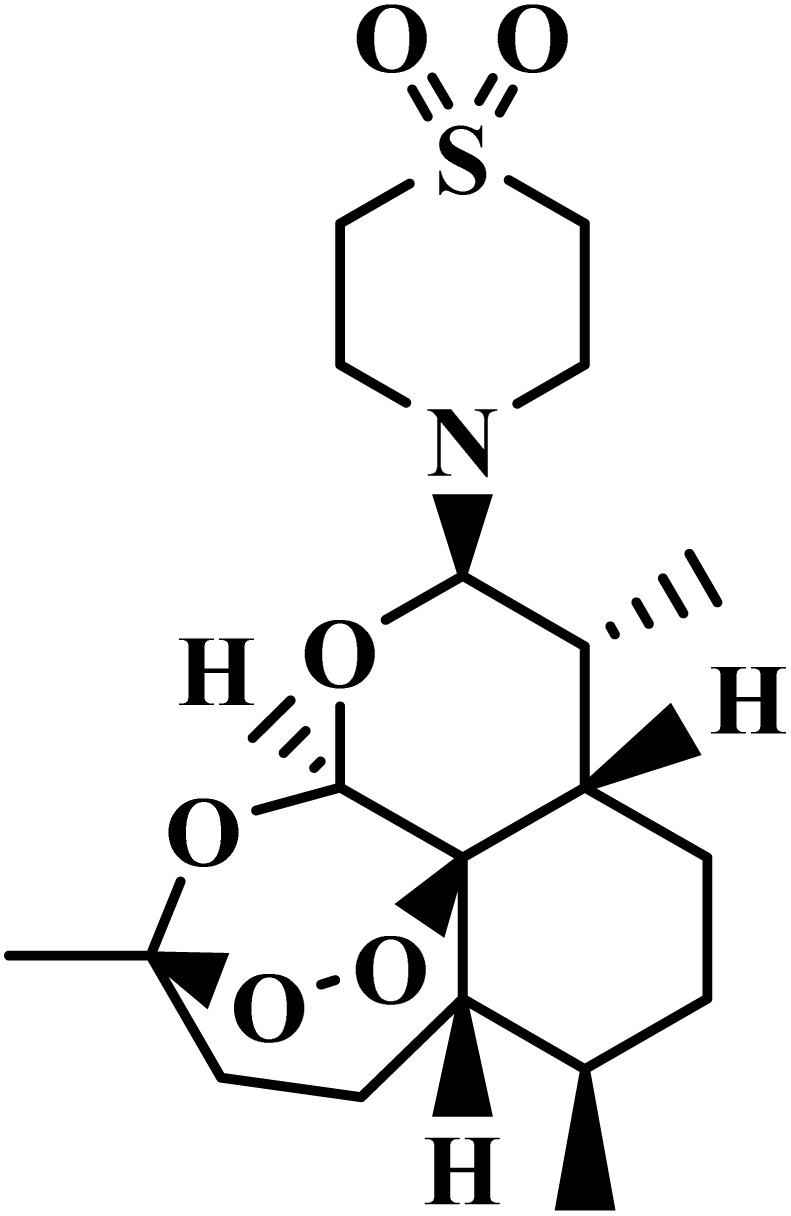	Artemisinin	III	Inhibits asexual stage and stage V gametocyte of *P. falciparum*^51^	(1) Low toxicity and non-neurotoxic profile^79,80^
					(2) Rapid metabolism with no detectable accumulation^79,80^
					(3) Generally well tolerated with no major adverse effects reported^81^
					(4) Safety established in adult males; further evaluation needed for children and pregnancy^81^
Fosmidomycin	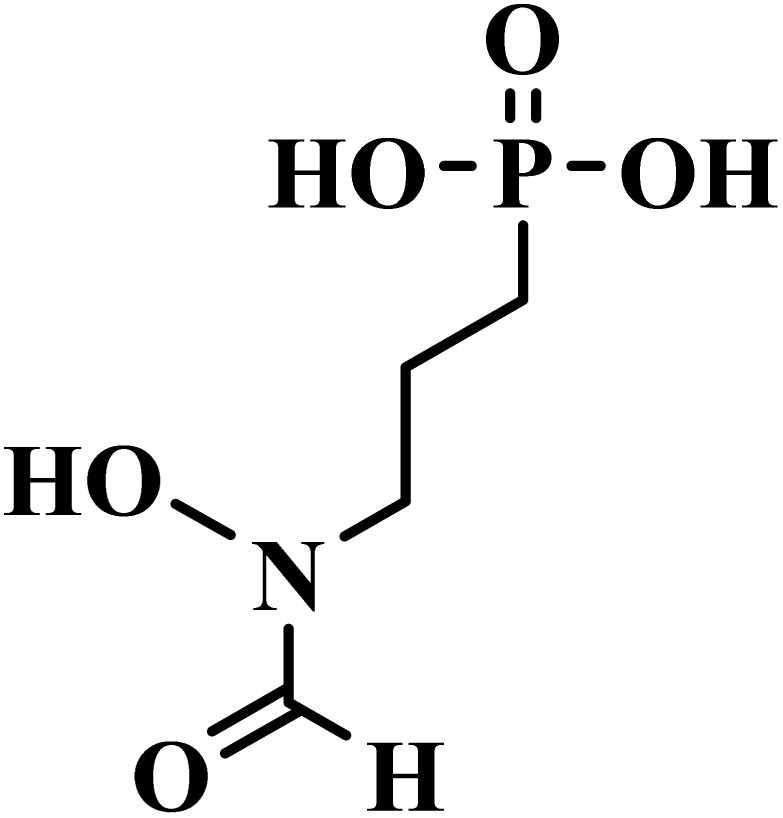	Phosphonic acid	III	Inhibits 1-deoxy-*d*-xylulose 5-phosphate reductoisomerase^51^	(1) Low toxicity due to selective inhibition of non-mevalonate pathway^82^
					(2) Generally well tolerated with no major adverse effects reported^51^
					(3) Safe for both children and adults^83^
Arterolane	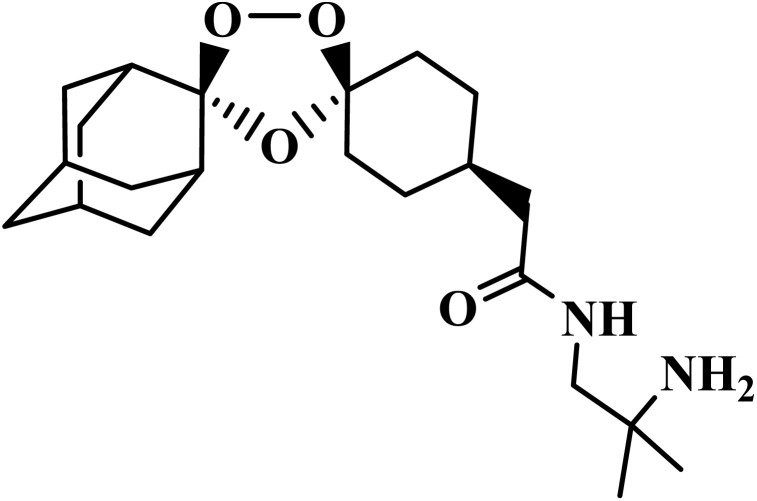	1,2,4-Trioxolane	III	Inhibits the detoxification of haem and *P. falciparum*-encoded sarcoplasmic endoplasmic reticulum calcium ATPase^51^	(1) Generally safe but may cause hematological effects (hyperkalemia, eosinophilia, anemia) when combined with piperaquine^84^
					(2) Mild effects: vertigo, abdominal pain, vomiting, and diarrhea^85^
					(3) Well tolerated in clinical trials, including combination therapy with piperaquine^86^
Tafenoquine	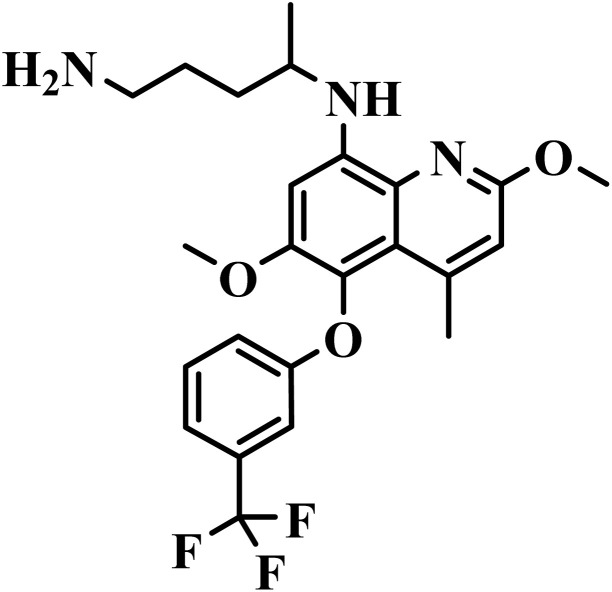	8-Aminoquinoline	IV	A prodrug that is activated in the liver by CYP2D6. No molecular targets have been yet identified.^87,88^	(1) Risk of hemolytic anemia, methemoglobinemia, and leukopenia in G6PD-deficient individuals^89^
					(2) Adverse effects include gastrointestinal symptoms, cardiac effects, neurological and psychiatric disturbances^90^
					(3) Long elimination half-life (2–3 weeks); generally safe for adults but contraindicated in G6PD deficiency^51^

## 2 Overview of FDA-approved antimalarial medications

### 2.1 Chloroquine (CQ)

**Fig. 3:**
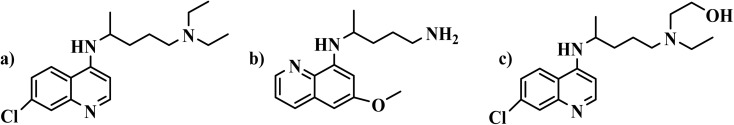
(a) Chloroquine; (b) Primaquine; (c) Hydroxychloroquine.

Chloroquine (Fig. 3a), a synthetic 4-aminoquinoline derivative of quinine, was approved by the FDA in 1949 as Aralen®, it became widely used by U.S. military personnel from 1950, often combined with primaquine for anti-relapse therapy.^10^ It passively diffuses into the *Plasmodium* parasite's digestive vacuole (DV), accumulating up to 5000 times higher than in uninfected RBCs due to the absence of a DV in mammalian cells. It binds to hematin, blocking its conversion to hemozoin, and causes toxic free heme buildup, disrupting parasite membranes and leading to death. It may also inhibit proteases involved in hemoglobin digestion, enhancing its efficacy.^14^ However, resistance has emerged in *P. falciparum* due to mutations in the *Pf*CRT gene.^15^ Resistance limits the parasite's ability to accumulate CQ in its digestive vacuole. It is ineffective against sporozoite and liver stages, offering minimal impact despite potential protection through sporozoite immunization and chemoprophylaxis.^16^ Common side effects include itching, gastrointestinal issues, headaches, skin rashes, and liver damage. Rarely, it can cause CNS toxicity, visual disturbances, fatigue, respiratory symptoms, and severe blood disorders like aplastic anemia, emphasizing the need for careful monitoring.^17^

### 2.2 Primaquine (PQ)

Primaquine (Fig. 3b) is a synthetic antimalarial drug belonging to the 8-aminoquinoline class that received FDA approval for military use in 1952, followed by civilian approval later that year.^10^ The drug is effective against *P. vivax* by targeting its gametocyte and hypnozoite stages. Once metabolized in the liver, it disrupts mitochondrial function, generates reactive oxygen species (ROS), and alters intracellular membranes, leading to oxidative damage in tissue-stage parasites.^18^ It has a low resistance rate, though some resistance in *P. vivax* blood stages has been observed. Its usual symptoms include methemoglobinemia (caused by overdose, with minimal cardiovascular toxicity), hemolysis in individuals with G6PD deficiency, and gastrointestinal discomfort, especially at higher doses. Rarely, it may cause neuropsychiatric effects such as depression and psychosis.^19^

### 2.3 Hydroxychloroquine (HCQ)

Hydroxychloroquine (Fig. 3c), a derivative of CQ was approved in the U.S. in 1955 as a 4-aminoquinoline, it is recognised for its safety and effectiveness and it is also listed among the World Health Organization's essential medicines due to its significant therapeutic value. The addition of a hydroxyl group makes HCQ less toxic and more soluble than CQ.^11^ HCQ disrupts lysosomal acidification, inhibiting key cellular processes such as proteolysis, chemotaxis, and antigen presentation. It also reduces pro-inflammatory cytokines (IL-1, IL-6), inhibits phospholipase A2, and counteracts inflammation. Additionally, it blocks UV-induced skin reactions, binds to DNA, and modulates immune responses by inhibiting calcium signaling in T and B cells. It also suppresses matrix metalloproteinases, preventing extracellular matrix degradation.^20^ However, its limitations include gastrointestinal issues, ocular toxicity that may cause vision impairment, and reduced efficacy in malaria prevention due to *Plasmodium* resistance, restricting its use in certain regions.^21^

### 2.4 Sulfadoxine-pyrimethamine

**Fig. 4:**
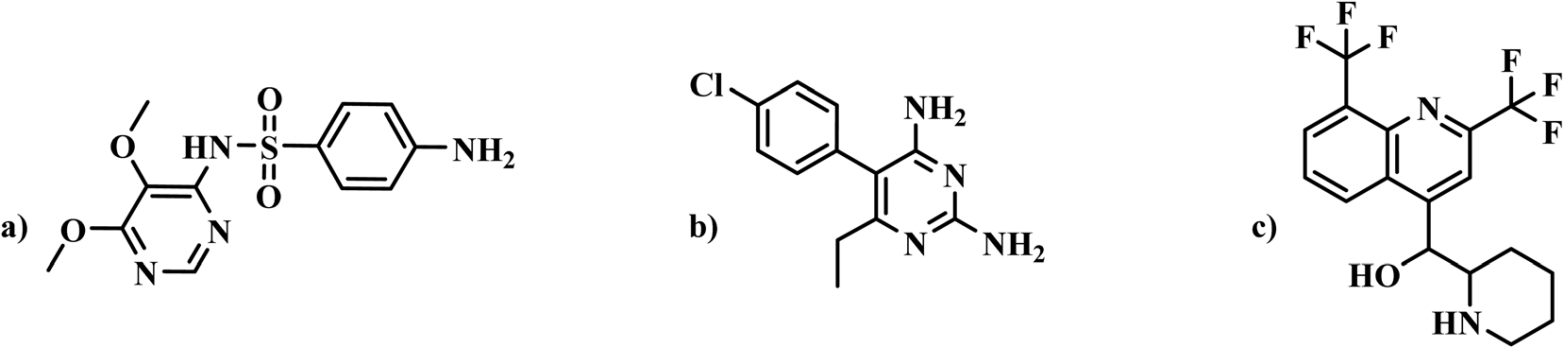
(a) Sulfadoxine; (b) Pyrimethamine; (c) Mefloquine.

Sulfa compounds were first used to treat malaria in the 1930s but declined due to resistance. Sulfadoxine (Fig. 4a) is one such sulfa compound. However, in the 1960s, the introduction of sulfadoxine-pyrimethamine revitalized malaria control efforts. Despite growing resistance, it remains effective, particularly in Africa.^6^ Sulfa drugs structurally resemble *p*-aminobenzoic acid (*p*ABA), and inhibit *Plasmodium* folate synthesis by disrupting the folate biosynthesis pathway. This lowers folate co-factors, which are essential for DNA and amino acid production. By inhibiting dihydropteroate synthase (DHPS), sulfa drugs impair thymidylate production and DNA synthesis, effectively targeting DHPS in multiple *Plasmodium* species.^22^

Pyrimethamine (Fig. 4b), a synthetic ethyl-pyrimidine derivative, inhibits the conversion of folic acid to folinic acid, which is essential for malaria parasite replication. By disrupting key metabolic reactions, it exerts a lethal effect on the parasite. When combined with sulphadiazine, its efficacy enhanced by targeting different points in the same pathway. Resistance to pyrimethamine is less common than proguanil, but can develop in the presence of *p*-aminobenzoic acid (*p*ABA), which antagonizes its action.^23^

The sulfadoxine–pyrimethamine combination is effective due to its dual inhibition of folic acid synthesis, enhancing anti-folic acid activity. Despite dapsone's severe unwanted effects, the sulfadoxine–pyrimethamine combination (Fansidar) received FDA approved in 1983 for malaria prevention.^6^ Resistance arise due to mutations in the *dhfr* gene (position 108) and *dhps* gene (multiple sites), reducing its efficacy.^24^ Severe adverse reactions to Fansidar include cholestatic hepatotoxicity, hepatic necrosis, hypersensitivity pneumonitis, and life-threatening skin conditions such as erythema multiforme, Stevens-Johnson syndrome, and toxic epidermal necrolysis.^25^

### 2.5 Mefloquine (MQ)

Mefloquine (Fig. 4c) was submitted for FDA approval in 1986 and approved in 1989.^10^ It targets cholinesterase enzymes, increasing neurotransmitter release, and disrupts membrane channels and ion transport, impairing neuronal communication. Additionally, it induces oxidative stress, affecting signaling proteins like Akt, which contributing to its cytotoxic effects.^26^ Its use is limited by resistance in *P. falciparum*, primarily due to mutations in the *pfmdr1* and *pfcrt* genes, which reduce drug accumulation and efficacy.^27^ MQ is also associated with neuropsychiatric complications, including anxiety, paranoia, and depression, especially with long-term use or in those with a history of epilepsy or depression. These effects are linked to enzyme inhibition and disrupted neuronal communication.

Despite these risks, MQ continues to be investigated for potential therapeutic applications, including the treatment of AIDS-related progressive multifocal leukoencephalopathy and autoimmune disorders.^28^

### 2.6 Atovaquone–proguanil

**Fig. 5:**
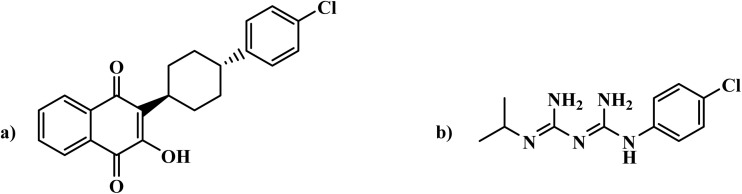
(a) Atovaquone; (b) Proguanil.

Atovaquone (Fig. 5a), a hydroxynaphthoquinone, inhibits the asexual erythrocytic stages of *Plasmodium* with an inhibitory concentration (IC50) range of 0.7 to 6 nM in studies.^29,30^ The drug inhibits mitochondrial electron transport by targeting the cytochrome bc1 complex, disrupting pyrimidine biosynthesis essential for parasite survival. *Plasmodium* relies on this pathway, whereas mammalian cells can salvage pyrimidines, making atovaquone selectively toxic to the parasite It also disrupts mitochondrial membrane potential in *P. falciparum* and *P. yoelii*.^31^ However, resistance can emerge due to mutations in the *cytochrome b* gene, particularly under drug pressure or when used as a monotherapy.^32^

Proguanil (Fig. 5b), a biguanide, is metabolized to cycloguanil, which effectively inhibits the asexual erythrocytic stages of *P. falciparum* with an IC50 range of 18 to 36 nM, while proguanil itself is less active.^33^ Cycloguanil selectively targets *Plasmodium* species by inhibiting dihydrofolate reductase (DHFR), depleting tetrahydrofolate cofactors, blocking DNA synthesis and halting parasite growth. Resistance to cycloguanil is linked to DHFR mutations, though proguanil retains some efficacy against resistant strains. Additionally, proguanil enhances atovaquone's effect by inducing mitochondrial toxicity.^34^

To address resistance concerns, a fixed-dose combination of atovaquone and proguanil, marketed as Malarone®, was approved in 2000 for malaria prevention and treatment.^10^ However, resistance to both drugs can develop through mitochondrial DNA mutations, particularly in the *cytochrome b* gene, while DHFR mutations may lead to cross-resistance with other antifolate drugs, complicating treatment.^35^ Despite these challenges, they have fewer neurological effects, such as anxiety, depression, and insomnia, compared to other antimalarials like MQ.^28^

### 2.7 Quinine

**Fig. 6:**
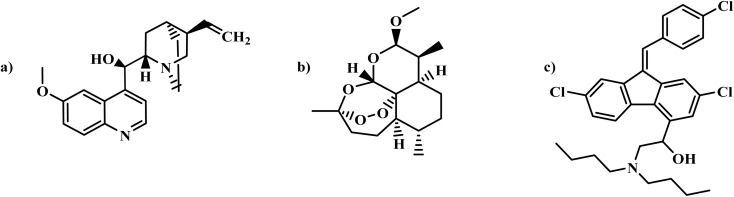
(a) Quinine; (b) Artemether; (c) Lumefantrine.

Quinine (Fig. 6a), approved by the FDA in 2005 for oral use, has never been approved for intravenous administration.^6^ It works by inhibiting heme polymerization and disrupting the parasite's detoxification of heme, although it has a weaker binding affinity compared to chloroquine. It impedes hemozoin formation, suppresses heme catalase activity, and interferes with the parasite's management of oxidative stress.^36^ Resistance to quinine develops due to mutations in the *Plasmodium falciparum* chloroquine resistance transporter (*PfCRT*) gene, which reduces drug accumulation in the parasite's vacuole and promotes active drug efflux. Resistance is further exacerbated by selective pressure and the emergence of multi-drug-resistant strains, complicating treatment efforts.^37^ It also has cognitive impairments, including tinnitus, anxiety, and sleep disturbances, caused by its inhibition of tryptophan hydroxylase (TPH2), which reduces serotonin production. These effects are intensified in low tryptophan environments, contributing to mood disturbances and depression. Despite its historical effectiveness, its efficacy has diminished due to resistance and side effects, underscoring the need for continuous research and monitoring.^38^

### 2.8 Artemether–lumefantrine

Artemether (Fig. 6b), derived from artemisinin in the *Artemisia annua* (sweet wormwood) plant, is highly effective against *P. falciparum*. It has a rapid onset of action and a short half-life of 2–3 hours, allowing for quick reduction of parasitemia, and rapid symptom relief.^39^ Its mechanism of action involves disrupting parasite transport proteins, impairing mitochondrial function, inhibiting angiogenesis, and modulating the host immune response.^40^

Lumefantrine (Fig. 6c), a vital component of antimalaria therapy, has a longer half-life of three to six days, ensuring sustained therapeutic drug levels over time.^41^ It is essential for treating uncomplicated *P. falciparum* malaria, acting as a blood schizonticide that targets the parasite during its blood stages. It disrupts heme conversion to hemozoin, leading to toxic heme accumulation and the parasite death.^42^

In 2009, the United States approved the fixed-dose combination artemether–lumefantrine (Coartem®) for treating uncomplicated malaria.^12^ The combination achieves cure rates over 95% in both adults and children. Artemether rapidly eliminates parasites, while lumefantrine's extended half-life sustains therapeutic levels, preventing resurgence. Additionally, the combination has gametocidal activity, helping reduce malaria transmission. Clinical evidence supports its safety, tolerability, and efficacy across all age groups, with no *in vivo* resistance reported in Africa, making it a primary treatment for malaria.^43^ However, resistance can develop due to genetic mutations, such as in the *K13-propeller* gene, as well as selective pressure from widespread use, inadequate dosing, or poor adherence. Parasites can adapt by altering drug targets, increasing drug efflux, and enhancing repair mechanisms, complicating treatment strategies.^44^ Ordinary aftereffects include headache, dizziness, nausea, and vomiting, with rare cases of severe allergic reactions. Its safety during pregnancy, especially in the first trimester, remains uncertain, requiring cautious use in pregnant and breastfeeding women.^37^

### 2.9 Tafenoquine (TQ)

**Fig. 7:**
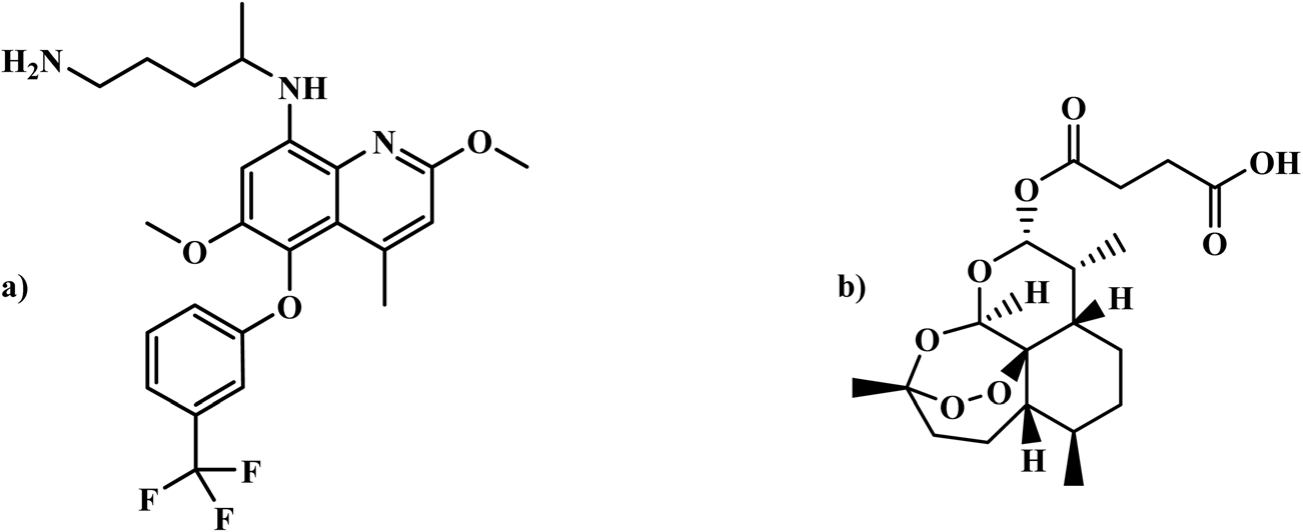
(a) Tafenoquine; (b) Intravenous Artesunate.

Tafenoquine (Fig. 7a), developed in 1978 by the Walter Reed Army Institute of Research, is a safer and more effective alternative to primaquine. In collaboration with GlaxoSmithKline and Medicines for Malaria Venture, the FDA approved Krintafel™ (150 mg) in July 2018 for the radical cure of *P. vivax* malaria. The following month, the FDA also approved Arakoda™ (100 mg) for malaria prophylaxis in individuals aged 18 and older, with permitted use for up to six months.^10^

It is metabolized by CYP 2D enzymes, generating reactive metabolites such as hydrogen peroxide, which induce oxidative stress in parasites. This disrupts cellular homeostasis, leading to mitochondrial dysfunction and increased intracellular calcium. Additionally, tafenoquine may produce radicals that alkylate parasite proteins and membrane lipids, further enhancing its antiparasitic effects.^45^ A major limitation of TQ is its potential to cause acute hemolytic anemia in G6PD-deficient patients, making it unsuitable for pregnant women, breastfeeding mothers, or individuals with unknown G6PD status. Commonly observed effects include gastrointestinal discomfort, headache, dizziness, and methemoglobinemia. Despite these concerns, TQ represents a significant advancement in malaria treatment and prevention.^46^

### 2.10 Intravenous artesunate (IV artesunate)

Artesunate (Fig. 7b), a semisynthetic derivative of artemisinin, is designed for improved solubility and absorption. It can be administered orally, rectally, intramuscularly, or intravenously to treat both uncomplicated and severe malaria. In May 2020, the FDA approved IV artesunate as a critical treatment for severe malaria.^13^ Artesunate works by inhibiting heme polymerization, generating reactive oxygen species (ROS), destabilizing parasite membranes, alkylating proteins, and inhibiting *Pf*ATP6. Its endoperoxide structure is activated by iron within the parasite, producing reactive intermediates that cause oxidative damage and parasite death. This activation occurs selectively in infected red blood cells, sparing uninfected cells.^47^

However, resistance to artesunate, particularly IV formulations, is emerging due to *kelch13* gene mutations. Additionally, IV administration requires trained personnel, limiting its accessibility in remote areas. Expected symptoms include allergic reactions and post-artesunate delayed hemolysis (PADH). Its high cost, limited availability, and need for monitoring further restrict widespread use, especially in resource-limited settings.^48^

## 3 Most promising antimalarial drugs in phase II, III and IV trials: towards FDA approval

The emergence of drug-resistant malaria underscores the urgent need for new antimalarial treatments. This section explores promising antimalarial candidates currently in clinical trials, emphasizing those with novel mechanisms and the potential FDA approval. These emerging therapies offer hope in enhancing treatment efficacy, overcoming resistance, and improving global malaria control efforts. Table 2 presents an overview of promising antimalarial drug candidates under various stages of clinical development, highlighting their chemical classes, mechanisms of action, safety profiles, and suitability for different patient populations. These candidates, including synthetic derivatives and repurposed drugs, target key pathways in *Plasmodium* survival and replication, such as ATP-dependent ion transport (*SJ733*), haemoglobin digestion inhibition (*Aretefenomel*, *Arterolane*), and *Pf*ATP4 inhibition (*Cipargamin*). Some, like M5717, act on novel targets such as the elongation factor 2 (*Pf*eEF2), offering new avenues for therapeutic intervention.

Safety remains a critical consideration in antimalarial drug development. Many candidates, such as Fosmidomycin and Artemisone, demonstrate favourable tolerability with minimal adverse effects, whereas others, such as Tafenoquine, require caution due to potential hemolytic anemia in G6PD-deficient individuals. Drugs like Ferroquine show efficacy but necessitate careful monitoring due to hepatotoxicity and cardiotoxicity risks. Additionally, some candidates, such as Rosiglitazone, offer an immunomodulatory role by enhancing macrophage-mediated parasite clearance, adding a complementary therapeutic dimension to traditional antiparasitic mechanisms.

The clinical pipeline spans phase II to IV trials, with some candidates advancing as monotherapies and others being explored in combination regimens to enhance efficacy and mitigate resistance development. Notably, Cipargamin and M5717 demonstrate rapid parasite clearance, making them strong candidates for further clinical advancement. The diversity of these compounds, spanning quinolines, spiroindolones, and phosphonic acids, underscores the ongoing efforts to develop next-generation antimalarial therapies. Continued research and clinical evaluation will be crucial in determining their long-term viability and potential integration into global malaria treatment strategies.

## 4 Drug targets for antimalarial drug discovery

One potential target for antimalarial therapies is the GPCR-like protein *PfSR25*, a potassium sensor that regulates calcium signaling within the *Plasmodium* parasite.^91^ Another promising target is *PfCRK4*, a cell-cycle regulator essential for DNA replication during schizogony, which plays a critical role in parasite growth and transmission.^92^

Enzymes involved in folate metabolism, such as thymidylate synthase (TS) and dihydrofolate reductase (DHFR), are crucial for the parasite's survival^93^ and are targeted by antifolate antimalarials like pyrimethamine and cycloguanil. Additionally, *PfDHODH*, an enzyme essential for pyrimidine production, is another key target in developing new treatments.^94^

*PfATP4*, an ATP-dependent sodium transporter, maintains low sodium levels by pumping sodium ions out of the parasite's cytoplasm, supporting survival within the erythrocyte by maintaining a favorable intracellular environment.^94^*PfATP4* also assists in hydrogen ion import, and acetyl–CoA synthetase is emerging as a novel target for future therapies.

The *Plasmodium* enzyme 1-deoxy-*d*-xylulose-5-phosphate reductoisomerase (*PfDXR*), involved in isoprenoid biosynthesis, is another promising candidate for new treatment development.^92^ Calcium-dependent protein kinases, like *PfCDPK7*, are crucial for the parasite's growth and development, making them potential targets for intervention. Additionally, *Plasmodium's* reliance on pyrimidine biosynthesis offers a promising avenue for therapeutic targeting.^95^

**Fig. 8:**
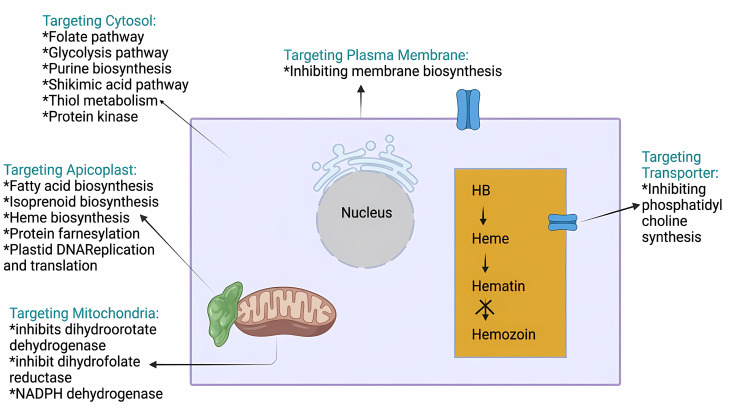
Drug targets for malaria.

Although *PfDHODH* is present in humans, its active site differs significantly from that of the parasite, allowing for the design of selective inhibitors that target the parasite without affecting the host.^92^ Other key targets include *PfPI4K*, essential for merozoite generation, and falcipains, which break down hemoglobin in the parasite's early stages.^96^ Despite the importance of falcipain-2 in the parasite's life cycle, efforts to develop drugs targeting this enzyme have faced challenges, possibly due to difficulty in avoiding the breakdown of host enzymes. Fig. 8 provides a summary of the key antimalarial targets and their modes of action.^97^

## 5 Regulatory landscapes and chemistry, manufacturing, and controls (CMC) considerations

The progression of an antimalarial lead compound from a laboratory to a clinically usable therapeutic is governed by strict rules and rigorous Chemistry, Manufacturing, and Controls (CMC) standards.^98^ Regulatory authorities, such as the U.S. Food and Drug Administration (FDA) and the European Medicines Agency (EMA), emphasize that the unique challenges associated with malaria like the need for treatment in tropical climates and the requirement for cheap, large-scale global distribution, necessitate particular attention to drug stability, reproducibility, and manufacturing scalability.^99,100^

### 5.1 FDA approval pathways and development incentives

The FDA issues product-specific guidance documents, such as the “*Malaria: Developing Drugs for Treatment*” guidance, which outlines regulatory expectations for clinical trial designs, efficacy endpoints, and safety assessments in both uncomplicated and severe malaria.^101^ To incentivize drug development for neglected tropical diseases, the FDA administers the Tropical Disease Priority Review Voucher (PRV) program under Section 524 of the Federal Food, Drug, and Cosmetic Act.^102^

This mechanism has been instrumental in the clinical translation of several key antimalarial therapies. For example, the 2009 approval of Artemether-Lumefantrine (Coartem) was associated with the first-ever PRV, and the 2018 approval of Tafenoquine (Krintafel) followed a similar incentive-driven pathway.^103^ These vouchers, which are transferable and provide a shortened six-month review timeline for subsequent, unrelated drug applications, play an essential role in bridging the gap between medicinal chemistry innovation and industrial investment.^102^

### 5.2 CMC and stability challenges

From a CMC perspective, the control of active pharmaceutical ingredient (API) quality and long-term stability represents a primary regulatory concern. This is especially critical for agents containing chemically sensitive motifs, such as the endoperoxide bridge in artemisinin derivatives.^104^

(a) Environmental stability (ICH Zone IVb): regulatory guidelines require stability assessments under climatic conditions representative of malaria-endemic regions, specifically International Council for Harmonisation (ICH) Zone IVb conditions (30 °C, 75% relative humidity). Ensuring that formulations remain potent without the need for cold-chain logistics is a critical CMC goal for deployment in resource-limited settings.^105^

(b) Polymorphism control: for lipophilic agents like Lumefantrine and newer clinical candidates like Cipargamin (KAE609), the control of crystal polymorphism during scale-up is essential. Variations in polymorphic forms can drastically alter dissolution behavior and oral bioavailability, directly impacting therapeutic efficacy. Strict CMC oversight is required to prevent sub-therapeutic dosing, which is a known driver for the development of parasite resistance.^106^

### 5.3 Large-scale manufacturing and advances in production strategies

A major advancement in antimalarial manufacturing has been the shift from agricultural extraction to semi-synthetic production routes. While the parent compound artemisinin is a natural product, it primarily serves as the starting material rather than final drug. Its derivatives, including artemether and IV artesunate, require high-purity intermediates.^107^

The industrialization of semi-synthetic artemisinin utilizes engineered *Saccharomyces cerevisiae* to produce artemisinic acid *via* fermentation. This acid is subsequently converted to artemisinin through a chemical photo-oxidation process. This state-of-the-art platform bypasses the seasonal volatility of *Artemisia annua* harvests, ensuring a consistent, high-purity supply for Artemisinin-based Combination Therapies (ACTs) and meeting the CMC requirements for global pharmaceutical distribution.^107^

## 6 Conclusions

Malaria, continues to pose a formidable challenge to global health despite decades of research and intervention. The detailed analysis provided here bridges the gap between FDA-approved antimalarial drugs and emerging therapeutic strategies, offering insights into their mechanisms of action, clinical successes, and inherent limitations. The recent arsenal of antimalarial drugs, from CQ to artemisinin-based combination therapies (ACTs), has significantly lowered the disease burden by targeting key parasite vulnerabilities such as mitochondrial function, folate biosynthesis, and heme detoxification. However, the rapid spread of drug-resistant *Plasmodium* strains, caused by mutations in genes like *PfCRT, dhfr,* and *K13*, threatens the efficacy of frontline treatments, stressing the urgent need for new therapeutic strategies.

Ongoing research is actively investigating new drug candidates such as artemisone, cipargamin, and SJ733, which employ innovative mechanisms like parasite metabolism disruption and *PfATP4* inhibition. Additionally, efforts to develop anti-relapse therapies, single-dose cures, and transmission-blocking agents, including primaquine and tafenoquine, are addressing critical challenges in malaria treatment. Combination therapies like atovaquone-proguanil and artemether-lumefantrine further highlight the benefits of synergistic approaches in strengthening treatment efficacy and combating resistance.

Optimizing antimalarial strategies to vulnerable populations remains essential, particularly for children, pregnant women, and individuals with glucose-6-phosphate dehydrogenase (G6PD) deficiency, who are at escalating risk of severe disease and treatment-related complications. Ensuring drug safety and minimizing adverse effects, such as neurological effects and hemolytic anemia, will be important for improving therapeutic outcomes.

Moving forward, integrating novel drug discovery with advanced clinical trials and improved resistance monitoring will be key to sustainable malaria control. Global health initiatives must prioritize equitable access to effective treatments, particularly in endemic regions, while strengthening public health infrastructure, vaccine development, and vector control to complement pharmacological advances in reducing the malaria burden.

## Author contributions

Monika drafted and wrote the review article. Bhawana Sharma conceptualized the study, contributed to the manuscript's structure, and finalized the content. Satish Kumar Awasthi provided supervision, critical insights, and overall guidance. All authors have read and approved the final version of the manuscript.

## Conflicts of interest

The authors declare no conflict of interest.

## Data Availability

No new data have been generated as part of this review.

## References

[cit1] Fikadu M., Ashenafi E. (2023). Infect. Drug Resist..

[cit2] World Health Organization , World Malaria Report 2024, WHO Press, Geneva, 2024

[cit3] Sato S. (2021). J. Physiol. Anthropol..

[cit4] Alharbi B. F., Ahmed M. A. (2026). Biology.

[cit5] Tse E. G., Korsik M., Todd M. H. (2019). Malar. J..

[cit6] Kitchen L. W., Vaughn D. W., Skillman D. R. (2006). Clin. Infect. Dis..

[cit7] Talapko J., Škrlec I., Alebić T., Jukić M., Včev A. (2019). Microorganisms.

[cit8] Dagen M. , in Antimalarial Agents, Elsevier, 2020, pp. 1–48

[cit9] Schlitzer M. (2007). ChemMedChem.

[cit10] Assessment of long-term health effects of antimalarial drugs when used for prophylaxis, ed. A. N. Styka and D. A. Savitz, National Academies of Sciences, Engineering, and Medicine, 2020 32369311

[cit11] Egbuna C. , Chandra S., Awuchi C. G., Saklani S., Ulhaq I., Akram M., Patrick-Iwuanyanwu K. C. and Khan J., in Coronavirus Drug Discovery, Elsevier, 2022, pp. 153–168

[cit12] Castro L., Ridpath A., Mace K., Gutman J. R. (2024). Clin. Infect. Dis..

[cit13] Thomas C. M., Stauffer W. M., Alpern J. D. (2023). Clin. Infect. Dis..

[cit14] Burgess S. J. , Design and Synthesis of Antimalarial Drugs Based on a Chloroquine Scaffold, Portland State University, 2008

[cit15] Wicht K. J., Mok S., Fidock D. A. (2020). Annu. Rev. Microbiol..

[cit16] Coban C. (2020). Curr. Opin. Immunol..

[cit17] Mulenga-Cilundika P., Ekofo J., Bagalwa D., Kabuya M., Chenge F. (2020). Int. J. Malar. Res. Rev..

[cit18] Foley M., Tilley L. (1998). Pharmacol. Therapeut..

[cit19] Vale N., Moreira R., Gomes P. (2009). Eur. J. Med. Chem..

[cit20] Ben-Zvi I., Kivity S., Langevitz P., Shoenfeld Y. (2012). Clin. Rev. Allergy Immunol..

[cit21] Stokkermans T. J. , Falkowitz D. M. and Trichonas G., in StatPearls [Internet], StatPearls Publishing, 2024 30725771

[cit22] Triglia T., Cowman A. F. (1999). Drug Resist. Updates.

[cit23] Rollo I. (1955). Br. J. Pharmacol. Chemother..

[cit24] Sibley C. H., Hyde J. E., Sims P. F., Plowe C. V., Kublin J. G., Mberu E. K., Cowman A. F., Winstanley P. A., Watkins W. M., Nzila A. M. (2001). Trends Parasitol..

[cit25] Peters P. J., Thigpen M. C., Parise M. E., Newman R. D. (2007). Drug Saf..

[cit26] Ghosh D. K., Kumar A., Ranjan A. (2021). Toxicology.

[cit27] Shafik S. H., Richards S. N., Corry B., Martin R. E. (2022). PLoS Biol..

[cit28] Grabias B., Kumar S. (2016). Expet Opin. Drug Saf..

[cit29] Gay F., Bustos D., Traore B., Jardinel C., Southammavong M., Ciceron L., Danis M. M. (1997). Am. J. Trop. Med. Hyg..

[cit30] Hudson A., Dickins M., Ginger C., Gutteridge W., Holdich T., Hutchinson D., Pudney M., Randall A., Latter V. (1991). Drugs Exp. Clin. Res..

[cit31] Srivastava I. K., Rottenberg H., Vaidya A. B. (1997). J. Biol. Chem..

[cit32] Pudney M., Gutteridge W., Zeman A., Dickins M., Woolley J. L. (1999). J. Trav. Med..

[cit33] Watkins W., Sixsmith D., Chulay J. (1984). Ann. Trop. Med. Parasitol..

[cit34] Fidock D. A., Wellems T. E. (1997). Proc. Natl. Acad. Sci. U. S. A..

[cit35] Cottrell G., Musset L., Hubert V., Le Bras J., Clain J. (2014). Antimicrob. Agents Chemother..

[cit36] Saifi M. A., Beg T., Harrath A. H., Altayalan F. S. H., Al Quraishy S. (2013). Afr. J. Pharm. Pharmacol..

[cit37] Antony H. A., Parija S. C. (2016). Trop. Parasitol..

[cit38] Islahudin F., Tindall S. M., Mellor I. R., Swift K., Christensen H. E., Fone K. C., Pleass R. J., Ting K.-N., Avery S. V. (2014). Sci. Rep..

[cit39] Byakika-Kibwika P., Lamorde M., Mayanja-Kizza H., Merry C., Colebunders B., Van Geertruyden J.-P. (2010). Therapeut. Clin. Risk Manag..

[cit40] Golenser J., Waknine J. H., Krugliak M., Hunt N. H., Grau G. E. (2006). Int. J. Parasitol..

[cit41] Travassos M. A., Laufer M. K. (2009). Pediatr. Res..

[cit42] Kokwaro G., Mwai L., Nzila A. (2007). Expet Opin. Pharmacother..

[cit43] Makanga M., Krudsood S. (2009). Malar. J..

[cit44] Djimde A. A., Makanga M., Kuhen K., Hamed K. (2015). Expert Rev. Anti-infect. Ther..

[cit45] Lu K.-Y., Derbyshire E. R. (2020). Biochemistry.

[cit46] Ashley E. A., Phyo A. P. (2018). Drugs.

[cit47] Ruwizhi N., Maseko R. B., Aderibigbe B. A. (2022). Pharmaceutics.

[cit48] Kouakou Y. I., Tod M., Leboucher G., Lavoignat A., Bonnot G., Bienvenu A.-L., Picot S. (2019). Int. J. Infect. Dis..

[cit49] Jiménez-Díaz M. B., Ebert D., Salinas Y., Pradhan A., Lehane A. M., Myrand-Lapierre M.-E., O'Loughlin K. G., Shackleford D. M., Justino de Almeida M., Carrillo A.
K. (2014). Proc. Natl. Acad. Sci. U. S. A..

[cit50] Gaur A. H., McCarthy J. S., Panetta J. C., Dallas R. H., Woodford J., Tang L., Smith A. M., Stewart T. B., Branum K. C., Freeman B. B. (2020). Lancet Infect. Dis..

[cit51] Umumararungu T., Nkuranga J. B., Habarurema G., Nyandwi J. B., Mukazayire M. J., Mukiza J., Muganga R., Hahirwa I., Mpenda M., Katembezi A. N. (2023). Bioorg. Med. Chem..

[cit52] Mengue J. B., Held J., Kreidenweiss A. (2019). Expet Opin. Invest. Drugs.

[cit53] Mzayek F., Deng H., Mather F. J., Wasilevich E. C., Liu H., Hadi C. M., Chansolme D. H., Murphy H. A., Melek B. H., Tenaglia A. N. (2007). PLoS Hub Clin. Trials.

[cit54] Berliner R. W., Earle D. P., Taggart J. V., Zubrod C. G., Welch W. J., Conan N. J., Bauman E., Scudder S. T., Shannon J. A. (1948). J. Clin. Investig..

[cit55] Goldgof G. M., Durrant J. D., Ottilie S., Vigil E., Allen K. E., Gunawan F., Kostylev M., Henderson K. A., Yang J., Schenken J. (2016). Sci. Rep..

[cit56] Yeung B. K., Zou B., Rottmann M., Lakshminarayana S. B., Ang S. H., Leong S. Y., Tan J., Wong J., Keller-Maerki S., Fischli C. (2010). J. Med. Chem..

[cit57] Kushwaha H., Misra A., Gautam N., Singh Y., Kumar H., Siddiqui H., Singh S. (2013). Drug Res..

[cit58] Nebot N., Crettol S., d'Esposito F., Tattam B., Hibbs D. E., Murray M. (2010). Br. J. Pharmacol..

[cit59] Bolton A. E., Peng B., Hubert M., Krebs-Brown A., Capdeville R., Keller U., Seiberling M. (2004). Cancer Chemother. Pharmacol..

[cit60] Dutreix C., Peng B., Mehring G., Hayes M., Capdeville R., Pokorny R., Seiberling M. (2004). Cancer Chemother. Pharmacol..

[cit61] Gambacorti-Passerini C., Antolini L., Mahon F.-X., Guilhot F., Deininger M., Fava C., Nagler A., Della Casa C. M., Morra E., Abruzzese E. (2011). J. Natl. Cancer Inst..

[cit62] Joensuu H., Trent J. C., Reichardt P. (2011). Cancer Treat Rev..

[cit63] O'Brien S. G., Guilhot F., Larson R. A., Gathmann I., Baccarani M., Cervantes F., Cornelissen J. J., Fischer T., Hochhaus A., Hughes T. (2003). N. Engl. J. Med..

[cit64] Jørgensen R., Merrill A. R., Andersen G. R. (2006). Biochem. Soc. Trans..

[cit65] Baragaña B., Hallyburton I., Lee M. C., Norcross N. R., Grimaldi R., Otto T. D., Proto W. R., Blagborough A. M., Meister S., Wirjanata G. (2015). Nature.

[cit66] Das A., Dash M. (2016). J. Emerging Infect. Dis..

[cit67] McCarthy J. S., Yalkinoglu Ö., Odedra A., Webster R., Oeuvray C., Tappert A., Bezuidenhout D., Giddins M. J., Dhingra S. K., Fidock D. A. (2021). Lancet Infect. Dis..

[cit68] Garavito G., Monje M.-C., Maurel S., Valentin A., Nepveu F., Deharo E. (2007). Exp. Parasitol..

[cit69] Lu G., Nagbanshi M., Goldau N., Mendes Jorge M., Meissner P., Jahn A., Mockenhaupt F. P., Mueller O. (2018). BMC Med..

[cit70] Müller O., Mockenhaupt F. P., Marks B., Meissner P., Coulibaly B., Kuhnert R., Buchner H., Schirmer R. H., Walter-Sack I., Sié A. (2013). Pharmacoepidemiol. Drug Saf..

[cit71] Varo R., Crowley V. M., Sitoe A., Madrid L., Serghides L., Bila R., Mucavele H., Mayor A., Bassat Q., Kain K. C. (2017). Malar. J..

[cit72] Varo R., Crowley V. M., Sitoe A., Madrid L., Serghides L., Kain K. C., Bassat Q. (2018). Malar. J..

[cit73] McCarthy J. S., Lotharius J., Rückle T., Chalon S., Phillips M. A., Elliott S., Sekuloski S., Griffin P., Ng C. L., Fidock D. A. (2017). Lancet Infect. Dis..

[cit74] Phillips M. A., Lotharius J., Marsh K., White J., Dayan A., White K. L., Njoroge J. W., El Mazouni F., Lao Y., Kokkonda S. (2015). Sci. Transl. Med..

[cit75] Adoke Y., Zoleko-Manego R., Ouoba S., Tiono A. B., Kaguthi G., Bonzela J. E., Duong T. T., Nahum A., Bouyou-Akotet M., Ogutu B. (2021). Malar. J..

[cit76] Charman S. A., Arbe-Barnes S., Bathurst I. C., Brun R., Campbell M., Charman W. N., Chiu F. C., Chollet J., Craft J. C., Creek D. J. (2011). Proc. Natl. Acad. Sci. U. S. A..

[cit77] Clark R. L., Edwards T. L., Longo M., Kinney J., Walker D. K., Rhodes J., Clode S. A., Rückle T., Wells T., Andenmatten N. (2018). Birth Defects Res..

[cit78] Moehrle J. J., Duparc S., Siethoff C., van Giersbergen P. L., Craft J. C., Arbe-Barnes S., Charman S. A., Gutierrez M., Wittlin S., Vennerstrom J. L. (2013). Br. J. Clin. Pharmacol..

[cit79] Haynes R. K., Fugmann B., Stetter J., Rieckmann K., Heilmann H. D., Chan H. W., Cheung M. K., Lam W. L., Wong H. N., Croft S. L. (2006). Angew. Chem., Int. Ed..

[cit80] Vivas L., Rattray L., Stewart L., Robinson B., Fugmann B., Haynes R., Peters W., Croft S. (2007). J. Antimicrob. Chemother..

[cit81] Nagelschmitz J., Voith B., Wensing G., Roemer A., Fugmann B., Haynes R. K., Kotecka B. M., Rieckmann K. H., Edstein M. D. (2008). Antimicrob. Agents Chemother..

[cit82] Yeh E., DeRisi J. L. (2011). PLoS Biol..

[cit83] Kuemmerle H., Murakawa T., Soneoka K., Konishi T. (1985). Int. J. Clin. Pharmacol. Ther. Toxicol..

[cit84] Valecha N., Krudsood S., Tangpukdee N., Mohanty S., Sharma S., Tyagi P., Anvikar A., Mohanty R., Rao B., Jha A. (2012). Clin. Infect. Dis..

[cit85] Valecha N., Looareesuwan S., Martensson A., Mohammed Abdulla S., Krudsood S., Tangpukdee N., Mohanty S., Mishra S. K., Tyagi P., Sharma S. (2010). Clin. Infect. Dis..

[cit86] Patil C., Katare S., Baig M., Doifode S. (2014). Ann. Med. Health Sci. Res..

[cit87] Vuong C., Xie L. H., Potter B. M., Zhang J., Zhang P., Duan D., Nolan C. K., Sciotti R. J., Zottig V. E., Nanayakkara N. D. (2015). Antimicrob. Agents Chemother..

[cit88] Vennerstrom J. L., Nuzum E. O., Miller R. E., Dorn A., Gerena L., Dande P. A., Ellis W. Y., Ridley R. G., Milhous W. K. (1999). Antimicrob. Agents Chemother..

[cit89] Nasveld P. E., Edstein M. D., Reid M., Brennan L., Harris I. E., Kitchener S. J., Leggat P. A., Pickford P., Kerr C., Ohrt C. (2010). Antimicrob. Agents Chemother..

[cit90] Hounkpatin A. B., Kreidenweiss A., Held J. (2019). Infect. Drug Resist..

[cit91] Wang P.-p., Jiang X., Zhu L., Zhou D., Hong M., He L., Chen L., Yao S., Zhao Y., Chen G. (2022). Microbiol. Spectr..

[cit92] Bagratee T., Prawlall R., Ndlovu T., Sibisi S., Ndadane S., Shaik B. B., Palkar M. B., Gampa R., Karpoormath R. (2024). Chem. Biodiversity.

[cit93] Kuesap J., Suphakhonchuwong N., Kalawong L., Khumchum N. (2022). J. Parasitol..

[cit94] Hoarau M., Vanichtanankul J., Srimongkolpithak N., Vitsupakorn D., Yuthavong Y., Kamchonwongpaisan S. (2021). J. Enzyme Inhib. Med. Chem..

[cit95] Kumar P., Tripathi A., Ranjan R., Halbert J., Gilberger T., Doerig C., Sharma P. (2014). J. Biol. Chem..

[cit96] Paquet T., Le Manach C., Cabrera D. G., Younis Y., Henrich P. P., Abraham T. S., Lee M. C., Basak R., Ghidelli-Disse S., Lafuente-Monasterio M. J. (2017). Sci. Transl. Med..

[cit97] Pandey K. C., Dixit R. (2012). J. Trop. Med..

[cit98] U. S. F. a. D. Administration, Chemistry, Manufacturing, and Controls (CMC) Guidances for Industry (GFIs) and Questions and Answers (Q&As), https://www.fda.gov/animal-veterinary/guidance-industry/chemistry-manufacturing-and-controls-cmc-guidances-industry-gfis-and-questions-and-answers-qas)

[cit99] E. M. A. (EMA) , The European Regulatory System for Medicines, European Medicines Agency, Amsterdam, The Netherlands, 2023

[cit100] U. S. F. a. D. Administration, Development & Approval Process (Drugs), https://www.fda.gov/drugs/development-approval-process-drugs, (accessed 10 Mar 2026)

[cit101] U. S. F. a. D. Administration, Malaria: Developing Drugs for Treatment—Draft Guidance for Industry, https://www.fda.gov/regulatory-information/search-fda-guidance-documents/malaria-developing-drugs-treatment)

[cit102] U. S. F. a. D. Administration, Tropical Disease Priority Review Voucher Program, https://www.fda.gov/about-fda/center-drug-evaluation-and-research-cder/tropical-disease-priority-review-voucher-program)

[cit103] Goh M., Outterson K., Kesselheim A. S. (2026). Health Afr. Sci..

[cit104] Bilia A., Morris G., Nilsson M. (2009). J. Pharmaceut. Sci..

[cit105] Organization W. H. , Requirements for Stability Studies of Finished Pharmaceutical Products, WHO Prequalification Team: Medicines, Geneva, 2010

[cit106] Clavier T. (2024). J. Chem. Pharm. Res..

[cit107] Kung S. H., Lund S., Murarka A., McPhee D., Paddon C. J. (2018). Front. Plant Sci..

